# Systematic Analysis of a Xenograft Mice Model for KSHV^+^ Primary Effusion Lymphoma (PEL)

**DOI:** 10.1371/journal.pone.0090349

**Published:** 2014-02-28

**Authors:** Lu Dai, Jimena Trillo-Tinoco, Lihua Bai, Baoli Kang, Zengguang Xu, Xiaofei Wen, Luis Del Valle, Zhiqiang Qin

**Affiliations:** 1 Research Center for Translational Medicine and Key Laboratory of Arrhythmias of the Ministry of Education of China, East Hospital, Tongji University School of Medicine, Shanghai, China; 2 Departments of Microbiology, Immunology & Parasitology, Louisiana Cancer Research Center, Louisiana State University Health Sciences Center, New Orleans, Louisiana, United States of America; 3 Department of Medicine, Louisiana Cancer Research Center, Louisiana State University Health Sciences Center, New Orleans, Louisiana, United States of America; 4 Department of Pathology, Louisiana Cancer Research Center, Louisiana State University Health Sciences Center, New Orleans, Louisiana, United States of America; 5 Central Laboratory, East Hospital, Tongji University School of Medicine, Shanghai, China; 6 Department of Urology, East Hospital, Tongji University School of Medicine, Shanghai, China; University of Southern California Keck School of Medicine, United States of America

## Abstract

Kaposi's sarcoma-associated herpesvirus is the causative agent of primary effusion lymphoma (PEL), which arises preferentially in the setting of infection with human immunodeficiency virus (HIV). Even with standard cytotoxic chemotherapy, PEL continues to cause high mortality rates, requiring the development of novel therapeutic strategies. PEL xenograft models employing immunodeficient mice have been used to study the *in vivo* effects of a variety of therapeutic approaches. However, it remains unclear whether these xenograft models entirely reflect clinical presentations of KSHV^+^ PEL, especially given the recent description of extracavitary solid tumor variants arising in patients. In addition, effusion and solid tumor cells propagated *in vivo* exhibit unique biology, differing from one another or from their parental cell lines propagated through *in vitro* culture. Therefore, we used a KSHV^+^ PEL/BCBL-1 xenograft model involving non-obese diabetic/severe-combined immunodeficient (NOD/SCID) mice, and compared characteristics of effusion and solid tumors with their parent cell culture-derived counterparts. Our results indicate that although this xenograft model can be used for study of effusion and solid lymphoma observed in patients, tumor cells *in vivo* display unique features to those passed *in vitro*, including viral lytic gene expression profile, rate of solid tumor development, the host proteins and the complex of tumor microenvironment. These items should be carefully considered when the xenograft model is used for testing novel therapeutic strategies against KSHV-related lymphoma.

## Introduction

The oncogenic γ-herpesvirus known as the Kaposi's sarcoma-associated herpesvirus (KSHV) is a principal causative agent of cancers arising in patients with compromised immune systems [1]. One of these cancers, primary effusion lymphoma (PEL), is composed of transformed B cells harboring KSHV episomes and arises preferentially within the pleural or peritoneal cavities of patients infected with HIV [2]. PEL typically presents with lymphomatous body cavity effusions in the absence of solid tumor masses. However, recent case reports have identified the co-existence of extracavitary solid variants of KSHV^+^ PEL in some patients.^3–5^ Regardless, PEL is a rapidly progressive malignancy, with a median survival of approximately 6 months even with standard chemotherapy [3]. Use of combination cytotoxic chemotherapies represents the standard approach for PEL [4], [5], but the toxicity of systemic chemotherapy synergizes with those caused by antiretroviral therapy or immune suppression, further limiting treatment efficacy [3], [4], [6].

Several novel approaches for PEL therapy that increase the survival for some patients have been reported in recent studies, but a lack of sufficient safety and efficacy data have precluded their routine use. In one case report, a patient with PEL treated with highly active antiretroviral therapy (HAART) for HIV infection and rituximab, an anti-CD20 monoclonal antibody, had a complete remission at one month [7], [8], but PEL cells generally do not express CD20 [9]. Additional experimental approaches under investigation include bortezomib, a proteasome inhibitor which inhibits NF-κB activation, and LY294002, an inhibitor of the PI3K/Akt pathway [10], [11]. Limited data also suggest that bortezomib works synergistically with cytotoxic chemotherapy [10]. The mTOR inhibitor rapamycin (sirolimus) inhibited PEL cell growth in a murine xenograft model [12], but mTOR inhibition with rapamycin paradoxically induces expression of the serine/threonine kinase Akt and tumor cell growth, resulting in treatment failures[13]. One recent study reports that a purine scaffold HSP90 inhibitor, BIIB021, selectively induces cell-cycle arrest and apoptosis for PEL, potentially through blocking NF-κB activation [14].

Currently, the lymphoma cell-lines established from patients with PEL harboring KSHV episomes represent a useful tool for study of viral pathogenesis and oncogenesis [15]. Several groups have developed PEL xenograft models in immunodeficient mice to study the effects of a variety of therapeutic strategies [12], [16]–[18]. In most studies, the PEL animal models are used to evaluate the therapeutic effects of pharmacological molecules, especially their influence of tumor growth *in vivo*. However, there is a lack of information regarding the biology of PEL cells associated with effusions and solid tumors in these models, and whether they exhibit similar biological characters to their parental cell lines passed *in vitro*, including viral gene expression and cell surface expression of traditional markers associated with their identification and/or function. In addition, it is important to understand how the xenograft animal models reflect the clinical presentations observed in PEL patients, especially the extracavitary solid lymphoma formation mentioned above. Therefore, here we used a KSHV^+^ PEL xenograft model, employing body cavity-based lymphoma cells (BCBL-1) and non-obese diabetic/severe-combined immunodeficient (NOD/SCID) mice, to characterize PEL cells from effusions and solid tumors in the model, and compared these cells with their counterparts passed in cell culture.

## Results

### Establishment of a PEL xenograft model in NOD/SCID mice

**Figure 1:**
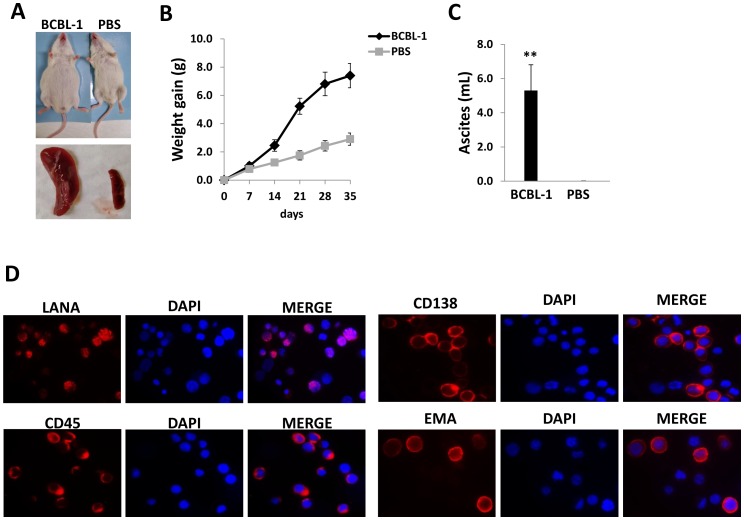
Establishment of a xenograft model for KSHV^+^ primary effusion lymphoma (PEL). (**A–C**) 10^7^ BCBL-1 cells or PBS controls were injected into the peritoneal cavity of NOD/SCID mice. Mice weight was recorded every week, 35 days later, mice were sacrificed and the volume of ascites fluid was measured. Error bars represent the S.E.M. for two experiments. ** =  p<0.01. (**D**) The ascites fractions were resuspended in PEL medium for 2 h in fibronectin-coated plates. Cells remaining in suspension were separated from adherent cells, and the expression of viral latent protein LANA, PEL-surface marker, CD45, CD138 and EMA was detected by immunofluorescence as described in Methods, respectively.

Intraperitoneal injection of KSHV^+^ BCBL-1 cells into the peritoneal cavity of 6–8 week-old male NOD/SCID mice resulted in rapid tumor growth, massive ascites and splenic enlargement within 3–4 weeks when compared with the PBS-injected control group (Fig. 1A-C). We confirmed PEL tumor expansion within the peritoneal compartment of the mice using immunofluorescence to identify the expression of representative phenotypic markers on the cell surface of ascites tumors, including CD45, CD138 and epithelial membrane antigen (EMA) (Fig. 1D). These markers are expressed on surface for most KSHV^+^ PEL cell lines isolated from patients, including BCBL-1 [6], [15]. We also confirmed the strong intranuclear expression of the KSHV-encoded latency-associated nuclear antigen (LANA) within ascites tumor cells, a unique marker for KSHV latently infected cells (Fig. 1D). After the dual staining, we actually found that ∼90%–95% LANA-positive cells were also positive for CD45, CD138 or EMA (data not shown). Together, these data demonstrate during the PEL tumor expansion *in vivo*, most tumor cells do not simultaneously lose viral episomes. In addition, we noticed that not all ascites cells expressed those phenotypic markers or LANA protein (∼70%–85% cells showing positive), potentially due to the cell populations contain a small fraction of immune cells, even after the purification procedure was performed as described in Methods.

### Immunological factors profile in tumor microenvironment

**Figure 2:**
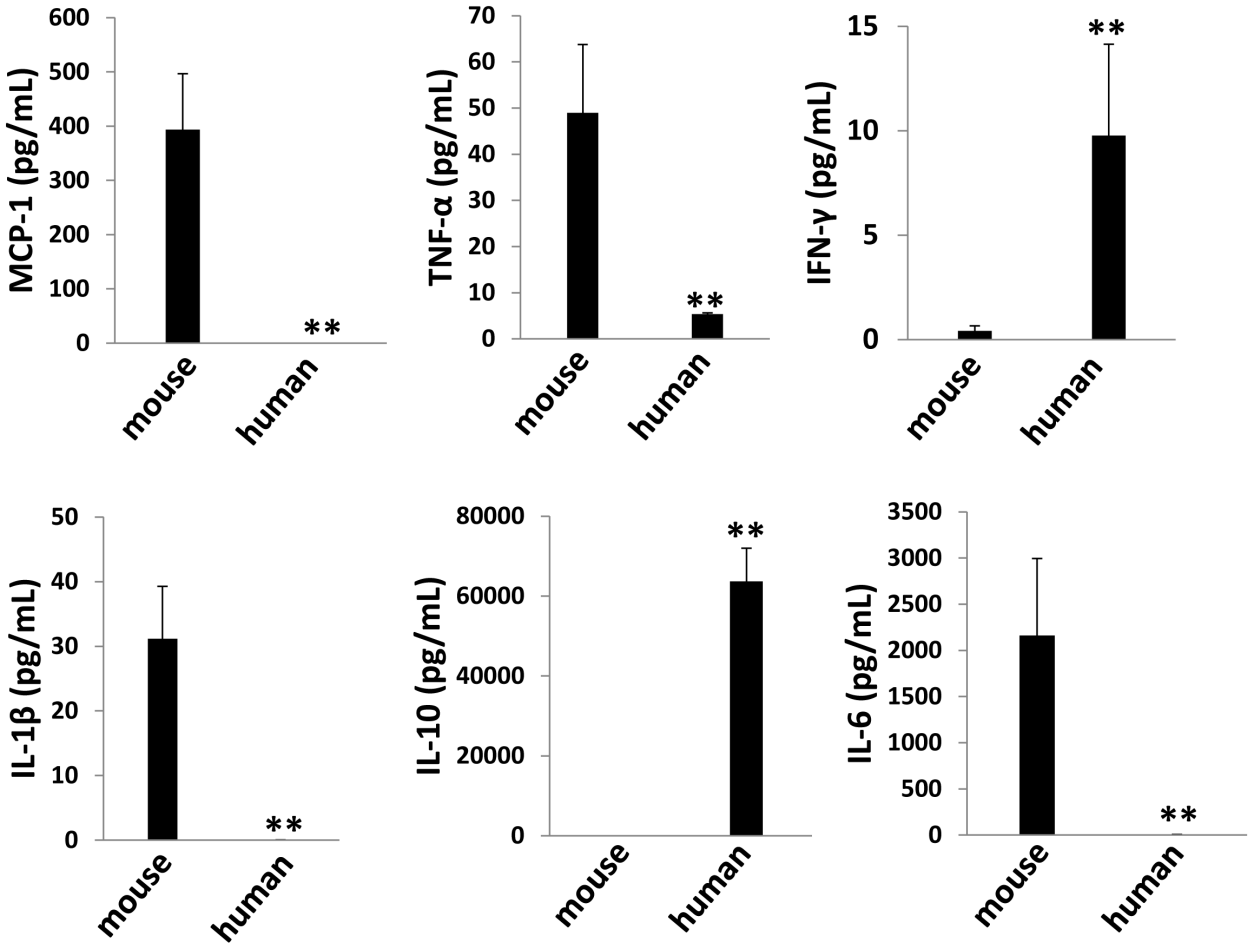
Cytokines production from ascites cells in NOD/SCID mice. Ascites fractions were collected from NOD/SCID mice as described above, then both human and mouse cytokines within the supernatant were measured by flow cytometry and ELISA as described in Methods. Error bars represent the S.E.M. for two experiments. ** = p<0.01.

Existing data suggest that some cytokines, such as IL-6, IL-10 and VEGF, play an important role in KSHV-related cancer pathogenesis, including the promotion of tumor cell growth, angiogenesis, and the suppression of T cell activation [19]–[22]. Especially, there are high levels of IL-6 and IL-10 in body cavity effusions from AIDS patients with PEL [20]. Therefore, we analyzed the profile of both human (h) and mouse (m) cytokines within the supernatant of ascites tumor directly isolated from mice, using flow cytometry and ELISA methods. In the tumor microenvironment of this xenograft model, we found the presence of both human (hIFN-γ and hIL-10) and mouse (mMCP-1, mIL-1β and mIL-6) immunological cytokines, while neither hIL-12p70 nor mIL-12p70 was detected in our samples. Notably, in each specific cytokine, only one species (human or mouse) derived was prominent in the tumor microenvironment (Fig. 2).

### Ascites tumor cells ex vivo display higher level of KSHV lytic gene expression than BCBL-1 in vitro culture

**Figure 3:**
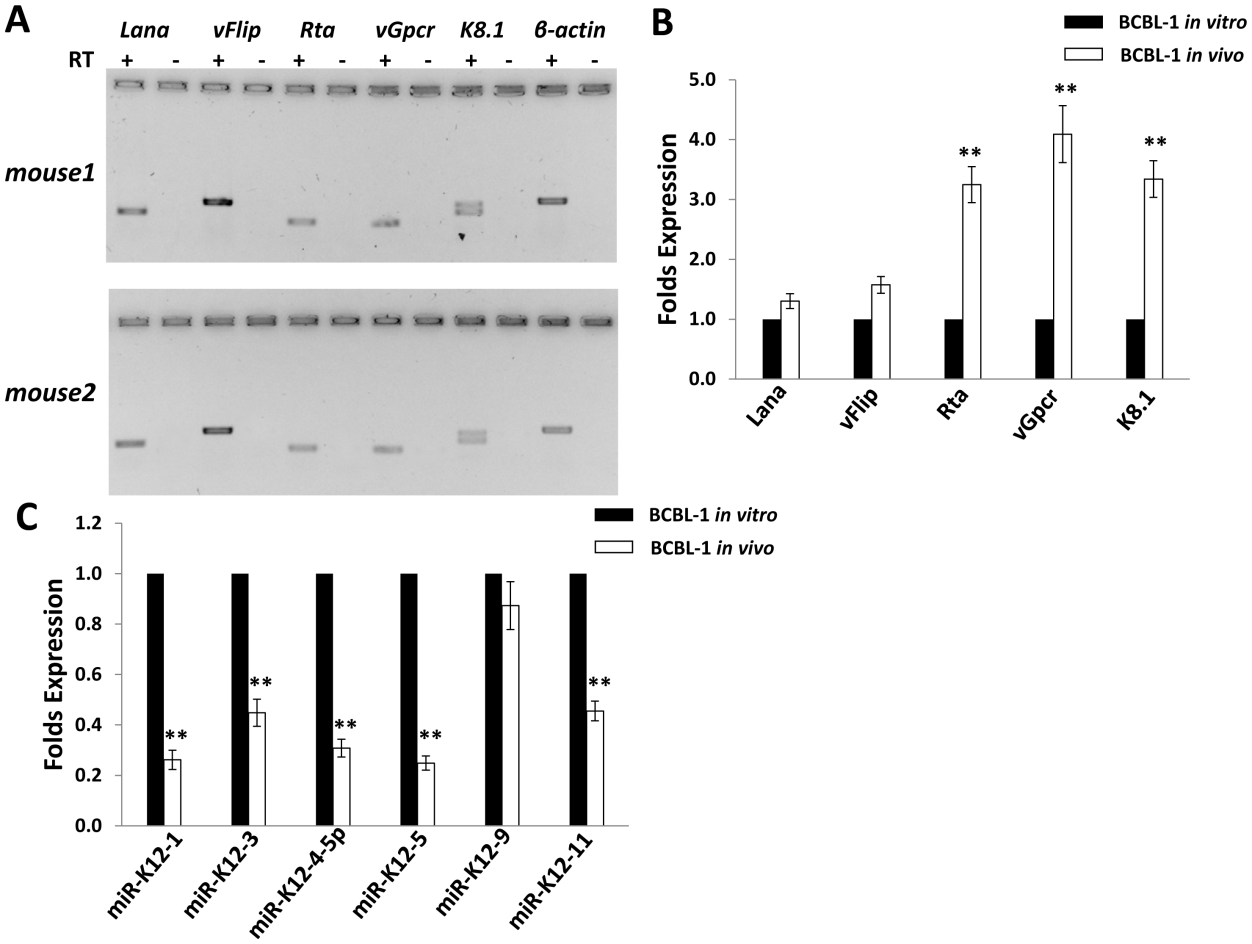
Expression profiling of KSHV viral genes from ascites cells in NOD/SCID mice. (**A**) Ascites fractions were collected and purified as described above, then expression of viral latent gene (*Lana, vFlip)* and lytic gene (*Rta, vGpcr, K8.1)* was detected by RT-PCR and agarose gel electrophoresis. *β-actin* was used as an internal control. (**B,C**) The expression of viral genes and microRNAs for *in vitro* cultured BCBL-1 and purified ascites cells from mice (shown as BCBL-1 *in vivo*) was compared by using qRT-PCR as described in Methods. Error bars represent the S.E.M for three experiments. ** = p<0.01.

As mentioned above, KSHV^+^ PEL cell-lines harbor viral episomes primarily in latent form, with only a very small minority of cells exhibiting linearized genomes and broader lytic gene expression [4]. Here, we first profiled expression of KSHV-encoded latent genes (*Lana, vFlip*) and lytic genes (*Rta, vGpcr, K8.1*), respectively, from ascites tumor cells of two mice using RT-PCR. Both latent and lytic genes transcripts were found detectable after the agarose gel electrophoresis (Fig. 3A). Next, we compared the profile of viral gene expression between ascites cells with BCBL-1 *in vitro* culture by qRT-PCR, and found that ascites cells displayed higher expression (∼3–5 folds) of lytic genes than BCBL-1 *in vitro* culture, while latent gene expression was slightly increased with no significance (Fig. 3B). One possible mechanism for inducing viral lytic genes is through repression of KSHV-encoded microRNAs (miRNAs). Thus far, 12 KSHV pre-miRNAs, encoding 18 mature miRNAs have been identified [23]–[25]. Recent published data demonstrate a role for KSHV miRNAs including miR-K12-1, 3, 4, 5, 9 and 11, in the regulation of viral “latent-lytic switch” in KSHV-infected cells (mostly for maintaining viral latency in host cells), through either direct targeting viral lytic reactivation activator, RTA [26], [27], or indirect mechanisms including targeting varied host factors including IκBα, nuclear factor I/B (NFIB) and IKKε [28]–[30]. Therefore, we sought to determine whether higher expressional levels of lytic genes in ascites cells were due to changes in the viral miRNA profile. Our results showed the reducing expression of most of viral miRNAs (miR-K12-1, 3, 4, 5 and 11), except miR-K12-9 in ascites cells when compared with BCBL-1 *in vitro* culture (Fig. 3C). Recent literatures report that oxidative stress or upregulation of reactive oxygen species (ROS) induces KSHV lytic reactivation from latently infected cells [31], [32]. Interestingly, our data indicated that there were higher levels of ROS within ascites supernatant than *in vitro* culture medium (Fig. S1), representing another potential mechanism for higher lytic gene expression within ascites cells. Our additional data indicated that there was a dramatic decreasing phosphorylation of NF-κB p65 kinase within ascites cells when compared with BCBL-1 *in vitro* culture (Fig. S2), which implies that the NF-κB pathway is probably involved in regulation of viral lytic gene expression and ROS production as described elsewhere [32].

### Solid tumors formed on various tissues and organs from the xenograft model

**Figure 4:**
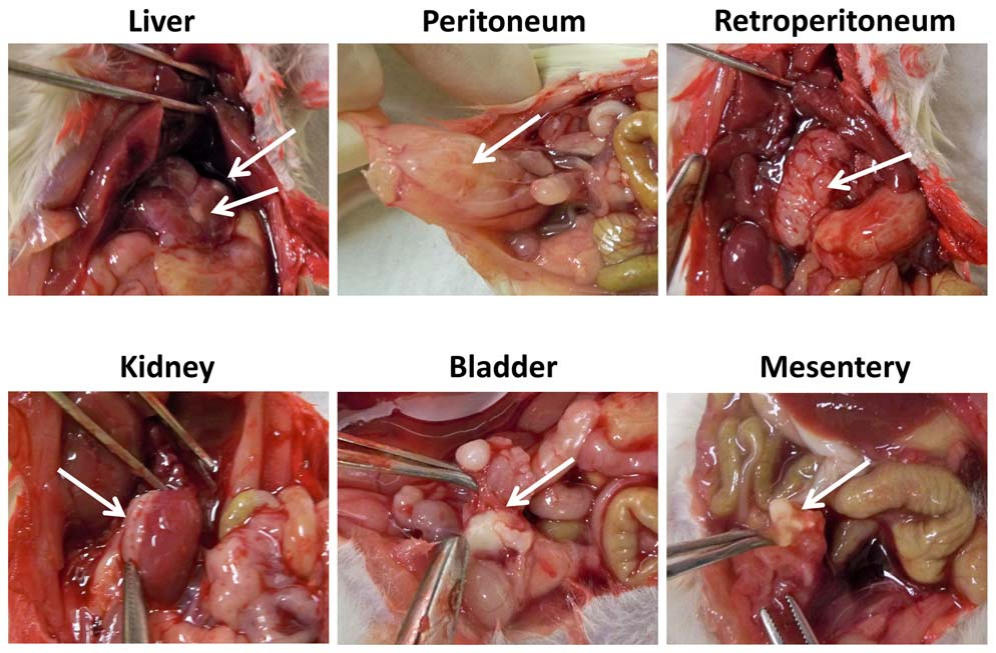
Solid tumors in a xenograft model for KSHV^+^ PEL. 10^7^ BCBL-1 cells were intraperitoneally injected into NOD/SCID mice, 42 days later, mice were sacrificed and the solid tumors (indicated by the arrows) were found on the multiple sites in mice.

As mentioned before, PEL usually presents as body cavity effusion but the absence of a solid tumor mass or recognizable nodal involvement [15]. However, recent studies have identified rare case reports of extracavitary solid variants of KSHV^+^ PEL in some tumor patients [3]–[5]. In our xenograft model, we found that ∼70–80% of mice formed single or multiple solid tumors on various tissues and organs (peritoneum, retroperitoneum, mesentery, liver, kidney and bladder as shown in Fig. 4), when sacrificed at 6 weeks after initial injection. In contrast to this, no mice formed visible solid tumors when sacrificed at 3 weeks after injection, while only ascites were found (data not shown). In addition, we did not observe tumor cells invading diaphragm and forming solid tumors on any tissues or organs such as lung in the thoracic cavity of our xenograft model, even sacrificed at 6 weeks post-injection.

### Comparison of viral and host protein profile between solid tumors and ascites

**Figure 5:**
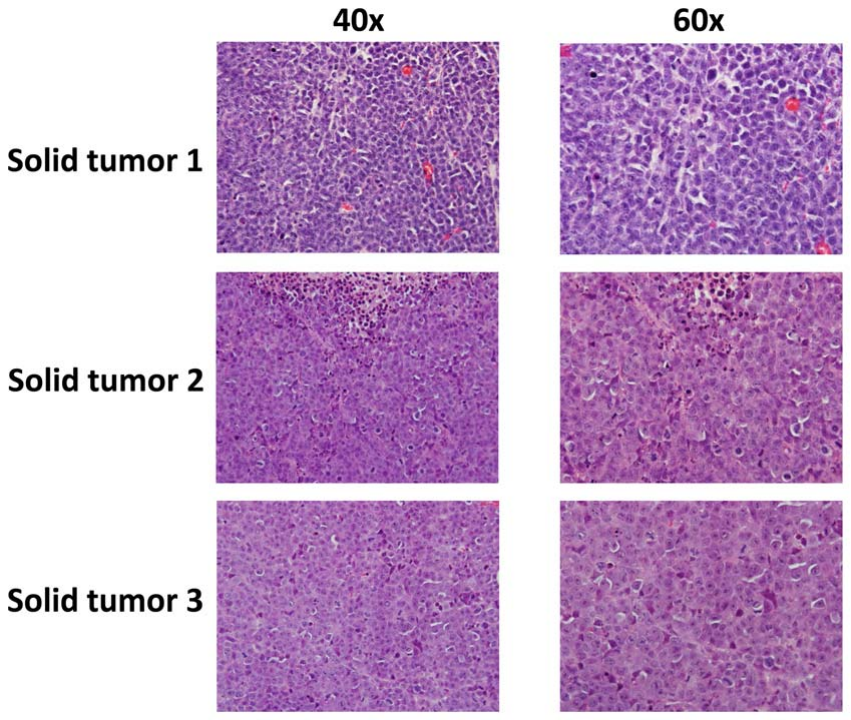
Characterization of the architecture of solid tumors in mice. Histological analysis of solid lymphoma isolated from 3 mice revealed an immunoblastic appearance, with sheaths of large, round cells with a large, chromatin extended nucleus and a prominent nucleolus. These cells show a high mitotic rate, areas of necrosis, and apoptosis in different stages, from picnotic nuclei to nuclear debris. (H&E stain; original magnification 400× for left panels and 600× for right panels).

**Figure 6:**
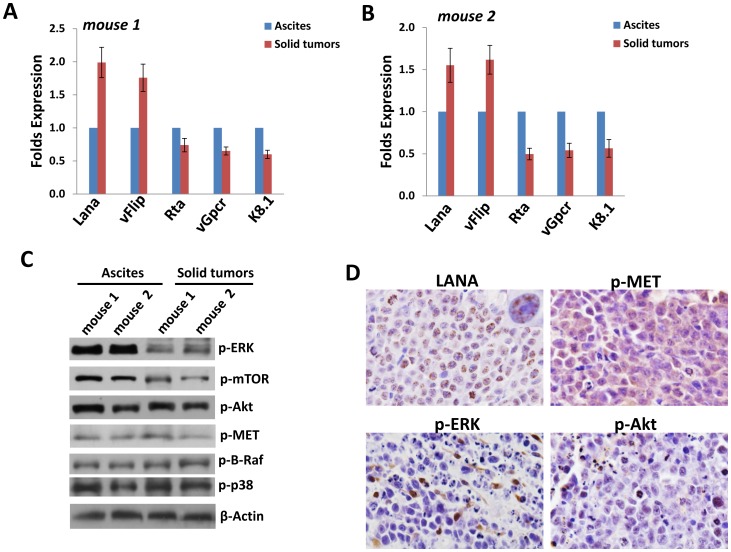
Comparison of viral and host proteins expression between solid tumors and ascites. (**A–B**) Total RNA was extracted from solid tumor tissues and ascites of 2 mice, then qRT-PCR was used to measure the expression of viral latent gene (*Lana, vFlip)* and lytic gene (*Rta, vGpcr, K8.1)*, respectively. Error bars represent the S.E.M for three experiments. (**C**) Immunoblots were used to detect proteins expression within solid tumor tissues and ascites samples. (**D**) Immunohistochemistry for LANA in solid lymphomas showed a characteristic punctate nuclear pattern in neoplastic cells. p-MET is strongly expressed in the cytoplasm of all tumor cells. p-ERK and p-Akt showed strong immunoreactivity only in a small number of malignant cells (Immunoperoxidase; original magnification for all panels is 1000×).

The solid tumor found in our xenograft model exhibited an immunoblastic appearance, with abundant to moderate amount of acidophilic cytoplasm with occasional perinuclear hofs, large nuclei with a single, prominent, centrally placed nucleolus, with a high mitotic rate, and were associated with a variable amount of apoptotic debris (Fig. 5). To compare the viral and host proteins expression between solid tumor tissues and ascites, we first used qRT-PCR to measure viral gene profile from 2 mice samples. Our results indicated the increased level of latent genes (*Lana, vFlip)* but reduced level of lytic gene (*Rta, vGpcr, K8.1)* within solid tumor tissues, when compared with ascites cells from the same mice (Fig. 6A–B). We also compared the viral gene profile between BCBL-1 in vitro culture and solid tumor tissues from mice, and found the higher levels of latent and lytic gene expression in solid tumors (Fig. S3). Immunoblots results confirmed the elevated LANA expression and reduced K8.1A expression in solid tumor tissues, when compared with ascites cells from the same mice (Fig. S4). For those host signaling transduction kinases which have been found essential for the survival and pathogenesis of PEL [11], [12], [32]–[34], solid tumor tissues displayed lower phosphorylation of MAPK-ERK and mTOR, while no significant change found in the phosphorylation of Akt, MET, B-Raf and MAPK-p38 kinases than those within ascites cells from the same mice (Fig. 6C). Finally to confirm viral and host proteins expression in the solid tumor tissue, we detected LANA, p-MET, p-ERK and p-Akt expression, respectively, using the immunohistochemistry methods. As shown in Fig. 6D, almost all solid tumor cells were strongly positive for intranuclear expression of LANA (brown dots), indicating that they were latently infected by KSHV. In addition, our results indicated that p-MET was strongly expressed in most cells of solid tumor samples, while p-ERK and p-Akt were only intermediately expressed in a few of solid tumor cells.

## Discussion

As mentioned above, immunological cytokines including IL-6, IL-10 play an important role in KSHV^+^ PEL development and pathogenesis in patients [19], [20]. Moreover, KSHV-encoded viral proteins such as K15, vGPCR (viral G protein-coupled receptor), vIL-6 (viral interleukin-6), and viral miRNAs have the capability of inducing a variety of cytokines production from host cells [35]-[38]. In our xenograft mice model, we found the presence of both human and mouse immunological cytokines within tumor microenvironment (Fig. 2), although their contributions to tumor development remain unclear. Therefore, several major questions need to be answered in future studies: 1) How these mouse-derived cytokines affect PEL tumor cells growth or expansion *in vivo* and whether they play similar roles as those human-derived cytokines homologous in patients? 2) Whether human-derived cytokines produced by PEL tumor cells have some affects to mouse body during tumor expansion? 3) It is interesting to measure the dynamics level of these cytokines during tumor development *in vivo*, which may help to identify their potential contribution to solid tumor formation as we observed. However, we think cautions need to be taken when comparing observations made in the xenografted NOD/SCID mice to clinical PEL, especially with regards to cytokines present in tumor microenvironment and solid tumor formation, as the severe immunological deficiencies in these mice may dramatically alter both their production of and response to the cytokine signaling.

In addition, PEL ascites tumor cells display higher level of lytic gene expression than those parental cells *in vitro* culture (Fig. 3). Based on this, we hypothesize that these lytic proteins may also contribute to tumor development in mice. Actually, several viral lytic proteins have been found important to KSHV^+^ PEL pathogenesis and cell growth [39]–[43]. For example, lytic protein vGPCR can activate ERK-2 and p38 pathways in PEL cells, which results in increased tumor cell elaboration of vIL-6 and VEGF, two growth factors involved in PEL pathogenesis [39]. Another lytic protein, vIL-6 and its cellular signal transducer gp130 are essential for PEL cell growth and survival *in vitro* culture [40], [41]. Interestingly, a recent study reported the inhibition of PEL engraftment *in vivo* by using morpholino oligomers against early lytic genes including vIL-6 [44]. Our additional data indicated that at least downregulation of KSHV-miRNAs which tend to act to maintain viral latency, and upregulation of ROS levels were involved in elevation of lytic gene expression in ascites, as described in previous PEL studies [23]–[32]. Other studies have reported that some host cytokines including oncostatin M, hepatocyte growth factor (HGF) and interferon-γ [45], [46]. However, we only found low level of interferon-γ present in ascites supernatant (Fig. 2), so whether these cytokines are really contributed to viral lytic genes reactivation *in vivo* still requires further experimental validation.

In our xenograft model, we found that 70–80% of mice finally formed single or multiple solid tumors on various tissues and organs (Fig. 4) at the end time-point of experiments, whose rate is much higher than that of PEL patients [3]–[5]. Interestingly, we do not notice solid tumor formation on any tissues or organs in the thoracic cavity in our xenograft model, although extracavitary solid lymphomas on chest wall and in lung of patients have been recorded in the literature [5], [47]. Cell morphology and KSHV/LANA-positive in nuclear confirmed that these solid tumors derived from injected PEL lymphoma cells, however, we noticed that solid tumor cells displayed the different viral and host proteins profile as ascites cells from the same mice. For example, phosphorylation of MAPK-ERK and mTOR were reduced in solid tumor tissues when compared with ascites cells (Fig. 6), while both of which are highly expressed and functional in PEL cells [12], [34] and implying they may have less contribution to solid tumor development and pathogenesis. Actually, a recent study has reported the distinctive gene and protein expression profile between effusion and solid lymphomas *in vivo*, using DNA microarray and proteomics analyses [48]. In details, a total of 14 proteins and 105 genes, respectively, whose expression differ significantly between effusion and solid lymphomas. However, due to the PEL cell-line and mouse species in that study are different from ours, the future work will analyze the gene and protein profile between ascites and solid lymphoma from our xenograft model to see whether we can get similar findings. In addition, it is important to understand whether there are different gene and protein profile between effusion and solid lymphoma samples directly from patients, although published data demonstrate no distinction between them based on morphology, immunophenotype and genotype [5].

Taken together, our data indicate that this xenograft model can be used for study of both effusion and solid lymphoma observed in PEL patients, which displays similar pathological feature such as morphology and immunophenotype. However, the complex of tumor microenvironment, the differential viral lytic gene expression, the rate of solid tumor development and the potential gene/protein sets differentiation of these solid tumor cells (including the contribution of host factors to oncogenesis) in mice etc, should be carefully considered when it is used for testing novel therapeutic strategies against KSHV-related lymphoma.

## Materials and Methods

### Cell culture and reagents

Body cavity-based lymphoma cells (BCBL-1, KSHV^+^/EBV^neg^) were originally purchased from ATCC (kindly provided by Dr. Dean Kedes, University of Virginia) and maintained in RPMI 1640 media (Gibco) supplemented with 10% fetal bovine serum (FBS), 10 mM HEPES (pH 7.5), 100 U/mL penicillin, 100 µg/mL streptomycin, 2 mM L-glutamine, 0.05 mM β-mercaptoethanol, and 0.02% (wt/vol) sodium bicarbonate.

### Ethics Statement

The animal protocols used in this study have been approved by the Louisiana State University Health Sciences Center (LSUHSC)-Institutional Animal Care and Use Committee.

### PEL xenograft model

6–8 week-old male NOD/SCID mice (Jackson Laboratory, Taconic Inc.) were maintained under specific pathogen-free conditions in LSUHSC animal care facility. Viable BCBL-1 cells from culture were washed twice in sterile-filtered PBS, and aliquots of 1×10^7^ cells were diluted in 200 µL PBS. The mice were received intraperitoneal (i.p.) injections with a single aliquot. Weights for individual mice were recorded weekly. Two experiments, with 10 mice per group for each experiment, were performed. For confirmation of PEL cell expansion within the murine model, ascites fractions from mice injected i.p. with BCBL-1 cells were removed 5–6 weeks after injection. Cells from each animal were resuspended in PEL media for 2 h in fibronectin-coated plates. Cells remaining in suspension were separated from adherent cells, and analyzed by immunofluorescence described below.

### PCR

Total RNA was isolated using the RNeasy Mini kit according to the manufacturer's instructions (QIAGEN). Solid tumor tissues were homogenized using the microtube pestles (Thomas Scientific) prior to add RLT lysis buffer provided in the RNeasy Mini kit. cDNA was synthesized from equivalent total RNA using SuperScript III First-Strand Synthesis SuperMix Kit (Invitrogen) according to the manufacturer's procedures (using oligo dT to prime RT reactions). Primers used for amplification of target genes are displayed in Table S1. Amplification was carried out using an iCycler IQ Real-Time PCR Detection System, and cycle threshold (Ct) values were tabulated in duplicate for each gene of interest in each experiment. “No template” (water) controls were used to ensure minimal background contamination. Using mean Ct values tabulated for each gene, and paired Ct values for β-actin as a loading control, fold changes for experimental groups relative to assigned controls were calculated using automated iQ5 2.0 software (Bio-rad). For amplification of viral miRNAs, cDNA was synthesized using the Taqman miRNA RT kit (Applied Biosystems), and qPCR was performed using the Taqman MicroRNA Assays kit (Applied Biosystems) and a 7500 Real Time PCR System. Target amplification for semi-quantitative PCR (RT-PCR) was performed using a DNA thermal cycler (Gene Amp PCR System 9700, Applied Biosystems) under conditions of 94°C for 5 min, 35 cycles of 94°C for 30 s, 54°C for 30 s, and 72°C for 60 s. Amplicons were subsequently identified by ethidium bromide-loaded agarose gel electrophoresis.

### Immunoblots

Ascites cells were lysed in buffer containing 20 mM Tris (pH 7.5), 150 mM NaCl, 1% NP40, 1 mM EDTA, 5 mM NaF and 5 mM Na_3_VO_4_. Solid tumor tissues were homogenized using the microtube pestles (Thomas Scientific) prior to add the lysis buffer above. Total cell lysates (30 µg) were resolved by 10% SDS–PAGE, transferred to nitrocellulose membranes, and incubated with 100–200 µg/mL of the following antibodies: phospho-Akt (Ser473), phospho-p44/42 ERK (Thr202/Tyr204), phospho-NF-κB p65 (Ser536), phospho-mTOR (Ser2448), phospho-B-Raf (Ser445), phospho-p38 MAPK (Thr180/Tyr182), phospho-Met (Tyr1234/1235) (Cell Signaling Technologies), KSHV-LANA (ABI) and K8.1A (Thermo Scientific-Pierce, Clone: 8C12G10G1). For loading controls, lysates were also incubated with antibodies detecting β-Actin (Sigma). Immunoreactive bands were developed using an enhanced chemiluminescence reaction (Perkin-Elmer).

### Immunohistochemistry

The formed tumors were dissected, fixed in formalin and embedded in paraffin. Sections of 4µm in thickness ere cut in a designated microtome, placed on electromagnetically charged slides (Fisher Scientific), and stained with Hematoxylin & Eosin (H&E) for routine histologic analysis. Immunohistochemistry was performed using the Avidin-Biotin-Peroxidase complex system, according to the manufacturer's instructions (Vectastain Elite ABC Peroxidase Kit; Vector Laboratories). In our modified protocol, sections were deparaffinized in xylene and re-hydrated through a descending alcohol gradient. For non-enzymatic antigen retrieval, slides were heated in 0.01 M sodium citrate buffer (pH 6.0) to 95°C under vacuum for 40 min and allowed to cool for 30 min at room temperature, then rinsed with PBS and incubated in MeOH/3% H_2_O_2_ for 20 min to quench endogenous peroxidase. Slides were then washed with PBS and blocked in PBS/0.1% BSA containing 5% normal rabbit serum (for rat monoclonal antibodies) or normal goat serum (for rabbit polyclonal antibodies) for 2 h at room temperature, then incubated overnight with primary anti-LANA rat monoclonal antibody (ABI) using a 1∶500 dilution, anti-p-Akt rabbit polyclonal antibody (Cell Signaling) using a 1∶500 dilution, anti-p-MET rabbit polyclonal antibody (Cell Signaling) using a 1∶1600 dilution and anti-p-ERK rabbit polyclonal antibody (Cell Signaling) using a 1∶400 dilution. The following day, slides were incubated with biotinylated secondary antibodies at room temperature for 1 h, followed by avidin-biotin peroxidase complexes for 1 h at room temperature. Finally, slides were developed using a diaminobenzidine substrate, counterstained with Hematoxylin, dehydrated through an ascending alcohol gradient, cleared in xylene, and coverslipped with Permount. Images were collected at 200× and 600× magnification using an Olympus BX300 inverted microscope equipped with a DP72 high resolution camera and CellSense image capture software.

### Immunofluorescence

Ascites tumor cells were incubated in 1∶1 methanol-acetone at −20°C for fixation and permeabilization, then with a blocking reagent (10% normal goat serum, 3% bovine serum albumin, and 1% glycine) for an additional 30 minutes. Cells were then incubated for 1 h at room temperature with 1∶1000 dilution of anti-LANA monoclonal antibody (ABI), or 1∶500 dilution of anti-CD138, anti-CD45 antibody (Santa Cruz), anti-EMA antibody (Thermo Fisher Scientific) followed by 1∶200 dilution of a goat anti-rat or goat anti-mouse secondary antibody conjugated to Texas Red (Invitrogen). For identification of nuclei, cells were subsequently counterstained with 0.5 µg/mL 4′,6-diamidino-2-phenylindole (DAPI; Sigma) in 180 mM Tris-HCl (pH 7.5). Slides were washed once in 180 mM Tris-HCl for 15 min and prepared for visualization using a Leica TCPS SP5 AOBS confocal microscope.

### Quantification of cytokines and extracellular reactive oxygen species (ROS)

Human/mouse cytokines, including IL-6, IL-10, MCP-1, IFN-γ, TNF-α, and IL-12p70, were quantified within ascites supernatants using the commercially available cytokine bead arrays (BD Biosciences) according to the manufacturer's instructions. Samples were analyzed using a Becton Dickinson FACSCalibur analytical flow cytometer and CellQuest Pro software (BD Biosciences). Human/mouse IL-1β was quantified using the commercially available ELISA kits (eBiosciences). The levels of ROS within ascites supernatants or conditioned medium were quantified using OxiSelect *In Vitro* ROS/RNS Assay Kit (Cell Biolabs) according to the manufacturer's instructions.

### Statistical analysis

Significance for differences between experimental and control groups were determined using the two-tailed Student's t-test (Excel 8.0) and p values <0.01 were considered significant.

## Supporting Information

Table S1 **Primer sequences for qRT-PCR or RT-PCR in this study.**
(DOCX)

Figure S1 **Elevated reactive oxygen species (ROS) level in the supernatant of ascites cells.** The extracellular ROS level within supernatant from *in vitro* cultured BCBL-1 and ascites fraction of mice (shown as BCBL-1 *in vivo*) was compared by using OxiSelect *In Vitro* ROS/RNS Assay Kit as described in Methods. Error bars represent the S.E.M for three experiments. ** = p<0.01.
(TIF)

Figure S2 **The NF-κB activities are dramatically decreased in ascites cells than BCBL-1** ***in vitro*** **culture.** Proteins were extracted from ascites of 2 mice and *in vitro* culture, then immunoblots were used to detect the protein expression. β-Actin was used as an internal control.
(TIF)

Figure S3 **Comparison of viral protein expression between BCBL-1** ***in vitro*** **culture and solid tumor tissues from mice.** Total RNA was extracted from solid tumor tissues of 3 mice or BCBL-1 *in vitro* culture, then qRT-PCR was used to measure the expression of viral latent gene (*Lana)* and lytic gene (*Rta, vGpcr, K8.1)*, respectively. Error bars represent the S.E.M for three experiments.
(TIF)

Figure S4 **Comparison of viral protein expression between solid tumor tissues and ascites.** Proteins were extracted from solid tumor tissues and ascites of 2 mice, and immunoblots were used to detect the expression of viral latent protein LANA and lytic protein K8.1A, respectively. β-Actin was used as an internal control.
(TIF)
