# Supplementary material for: Systematic Analysis of a Xenograft Mice Model for KSHV+ Primary Effusion Lymphoma (PEL)
Source: PLoS One. 2014 Feb 28;9(2):e90349. doi: 10.1371/journal.pone.0090349 (PMC3938717; doi:10.1371/journal.pone.0090349)
Supplement: Table S1 — Primer sequences for qRT-PCR or RT-PCR in this study. (DOCX) [file pone.0090349.s001.docx]

**Supplemental Table 1. Primer sequences for qRT-PCR or RT-PCR in this study.**

| **Gene** | **Sequences (5’ 3’)** |
| --- | --- |
| *Lana* | *sense TCCCTCTACACTAAACCCAATA*  *antisense TTGCTAATCTCGTTGTCCC* |
| *vFlip* | *sense GGGCACGGATGACAGGGAA*  *antisense TGTGATGGGCCGGAAAGG* |
| *Rta* | *sense TAATGTCAGCGTCCACTCC*  *antisense TTCTGGCACGGTCAAAGC* |
| *vGpcr* | *sense CATCCGCTGCACTGTTAA*  *antisense GCTTTGTCCTCCTCACCA* |
| *K8.1* | *sense CACCACAGAACTGACCGATG*  *antisense TGGCACACGGTTACTAGCAC* |
| *β-actin* | *sense GGAAATCGTGCGTGACATT*  *antisense GACTCGTCATACTCCTGCTTG* |
