# Supplementary figures and images for: Systematic Analysis of a Xenograft Mice Model for KSHV+ Primary Effusion Lymphoma (PEL)
Source: PLoS One. 2014 Feb 28;9(2):e90349. doi: 10.1371/journal.pone.0090349 (PMC3938717; doi:10.1371/journal.pone.0090349)

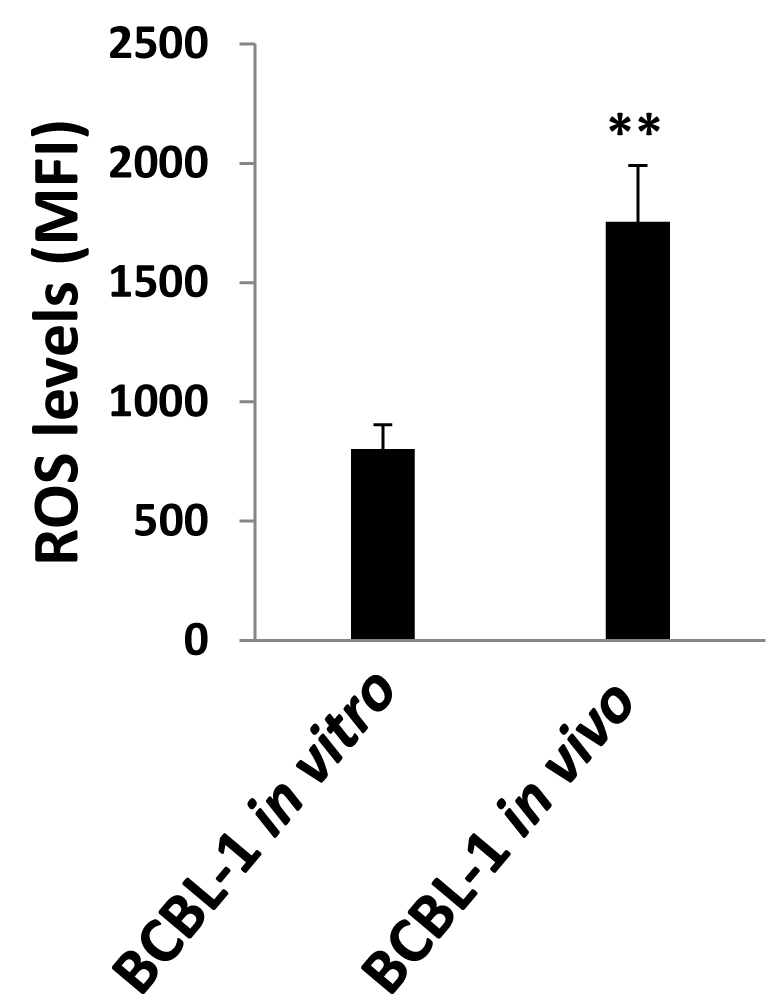

Supplement: Figure S1 — Elevated reactive oxygen species (ROS) level in the supernatant of ascites cells. The extracellular ROS level within supernatant from in vitro cultured BCBL-1 and ascites fraction of mice (shown as BCBL-1 in vivo) was compared by using OxiSelect In Vitro ROS/RNS Assay Kit as described in Methods. Error bars represent the S.E.M for three experiments. ** = p<0.01. (TIF) [file pone.0090349.s002.tif]

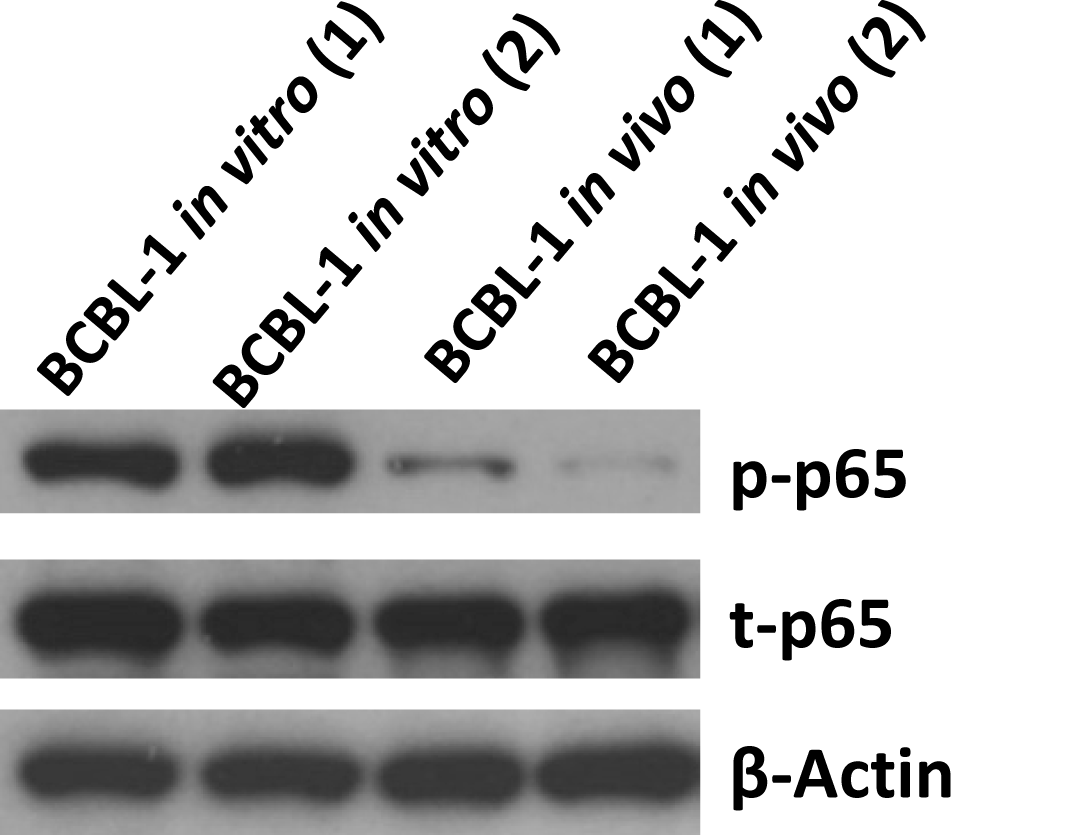

Supplement: Figure S2 — The NF-κB activities are dramatically decreased in ascites cells than BCBL-1 in vitro culture. Proteins were extracted from ascites of 2 mice and in vitro culture, then immunoblots were used to detect the protein expression. β-Actin was used as an internal control. (TIF) [file pone.0090349.s003.tif]

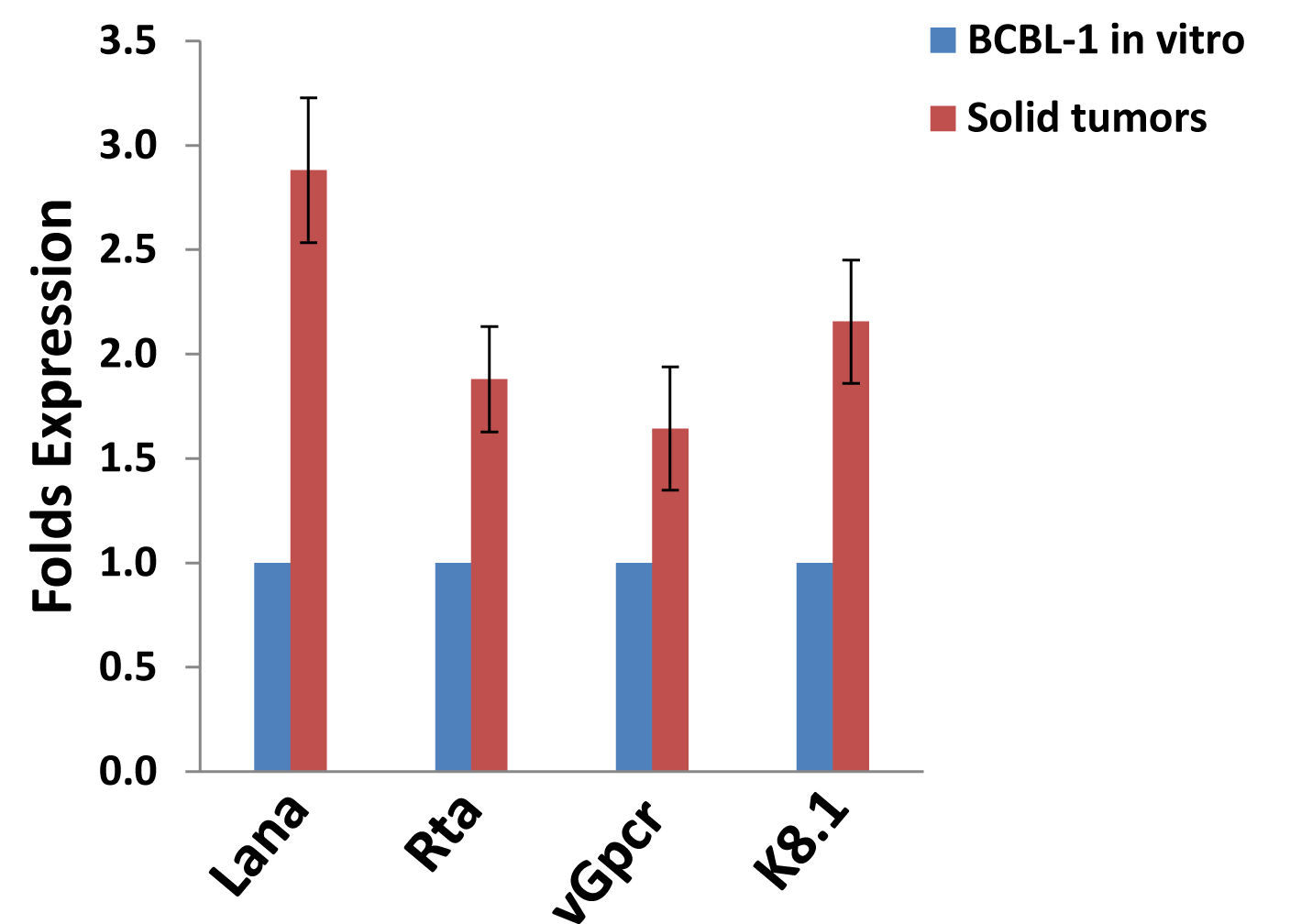

Supplement: Figure S3 — Comparison of viral protein expression between BCBL-1 in vitro culture and solid tumor tissues from mice. Total RNA was extracted from solid tumor tissues of 3 mice or BCBL-1 in vitro culture, then qRT-PCR was used to measure the expression of viral latent gene (Lana) and lytic gene (Rta, vGpcr, K8.1), respectively. Error bars represent the S.E.M for three experiments. (TIF) [file pone.0090349.s004.tif]

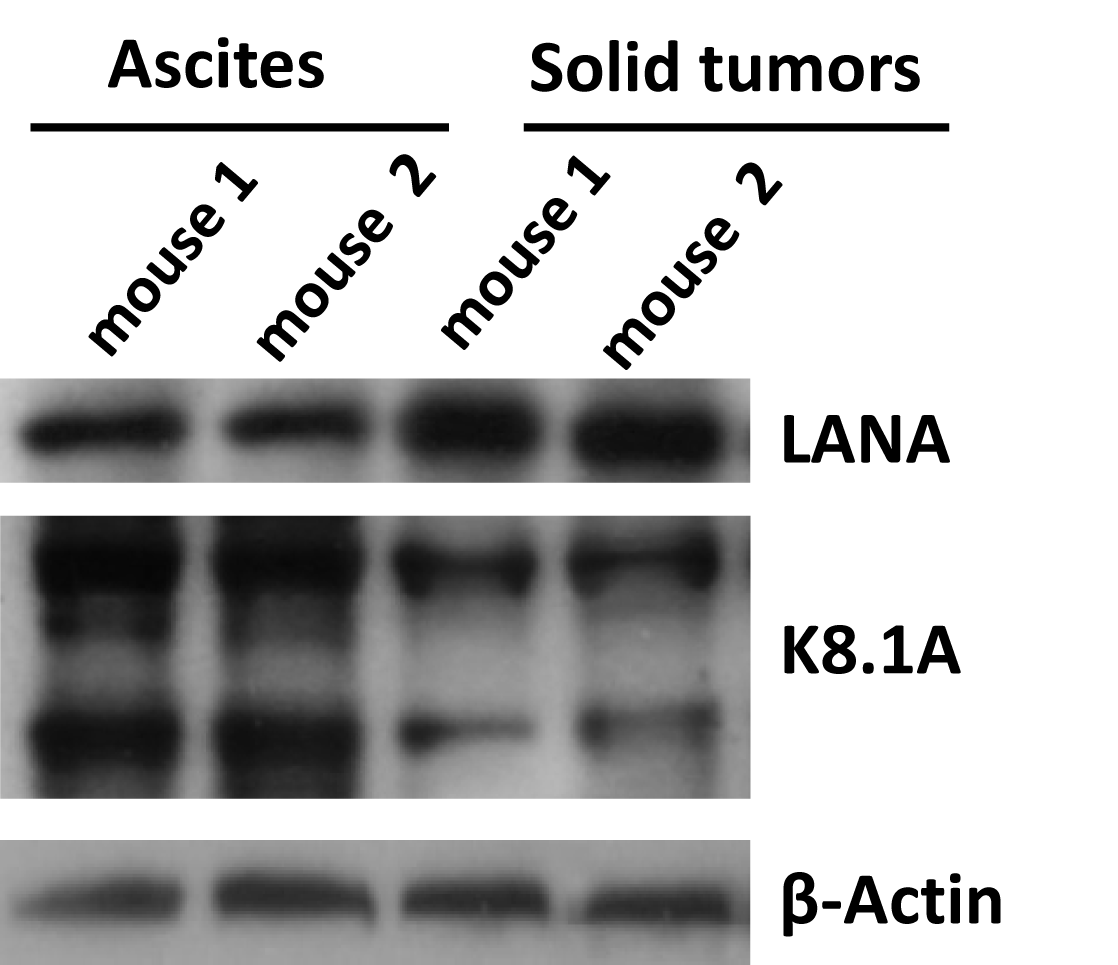

Supplement: Figure S4 — Comparison of viral protein expression between solid tumor tissues and ascites. Proteins were extracted from solid tumor tissues and ascites of 2 mice, and immunoblots were used to detect the expression of viral latent protein LANA and lytic protein K8.1A, respectively. β-Actin was used as an internal control. (TIF) [file pone.0090349.s005.tif]
